# Returning to dialysis after kidney allograft failure: the experience of the Italian Registry of Paediatric Chronic Dialysis

**DOI:** 10.1007/s00467-021-05140-6

**Published:** 2021-06-14

**Authors:** Edoardo La Porta, Ester Conversano, Daniela Zugna, Roberta Camilla, Raffaella Labbadia, Fabio Paglialonga, Mattia Parolin, Enrico Vidal, Enrico Verrina, on behalf of the Italian Registry of Paediatric Chronic Dialysis

**Affiliations:** 1 Dialysis Unit, Department of Pediatrics, IRCCS Giannina Gaslini, Genova, Italy; 2 Department of Pediatrics, Institute for Maternal and Child Health-IRCCS Burlo Garofolo, Trieste, Italy; 3 Department of Medical Sciences, Cancer Epidemiology Unit, University of Torino and CPO-Piemonte, Turin, Italy; 4 Paediatric Nephrology Unit, Regina Margherita Children’s Hospital, CDSS, Turin, Italy; 5 Nephrology and Dialysis Unit, Department of Pediatrics, “Bambino Gesù” Children’s Hospital-IRCCS, Rome, Italy; 6 Pediatric Nephrology, Dialysis and Transplant Unit, Fondazione IRCCS Ca’ Granda Ospedale Maggiore Policlinico, Milan, Italy; 7 Pediatric Nephrology, Dialysis and Transplant Unit, Department of Woman’s and Child’s Health, University Hospital, Padova, Padua, Italy; 8 Division of Pediatrics, Department of Medicine (DAME), University of Udine, P.le S.M della Misericordia, 15 33100, Udine, Italy

**Keywords:** Paediatric dialysis, Kidney transplantation, Kidney allograft failure, Complications, Death

## Abstract

**Background:**

The need for dialysis after kidney allograft failure (DAGF) is among the top five reasons for dialysis initiation, making this an important topic in clinical nephrology. However, data are scarce on dialysis choice after transplantation and clinical outcomes for DAGF in children.

**Methods:**

Patients receiving chronic dialysis < 18 years were recorded from January 1991 to January 2019 by the Italian Registry of Pediatric Chronic Dialysis (IRPCD). We investigated factors influencing choice of dialysis modality, patient outcome in terms of mortality, switching dialysis modality, and kidney transplantation.

**Results:**

Among 118 patients receiving DAGF, 41 (35%) were treated with peritoneal dialysis (PD), and 77 (65%) with haemodialysis (HD). Significant predictors for treatment with PD were younger age at dialysis start (OR 0.85 per year increase [95%CI 0.72–1.00]) and PD use before kidney transplantation (OR 8.20 [95%CI 1.82–37.01]). Patients entering DAGF in more recent eras (OR 0.87 per year increase [95%CI 0.80–0.94]) and with more than one dialysis modality before kidney transplantation (OR 0.56 for being treated with PD [0.12–2.59]) were more likely to be initiated on HD. As compared to patients on HD, those treated with PD exhibited increased but non-significant mortality risk (HR 2.15 [95%CI 0.54–8.6]; p = 0.28) and higher prevalence of dialysis-related complications during DAGF (p = 0.002)

**Conclusions:**

Patients entering DAGF in more recent years are more likely to be initiated on HD. In this specific population of children, use of PD seems associated with a more complicated course.

**Graphical abstract:**

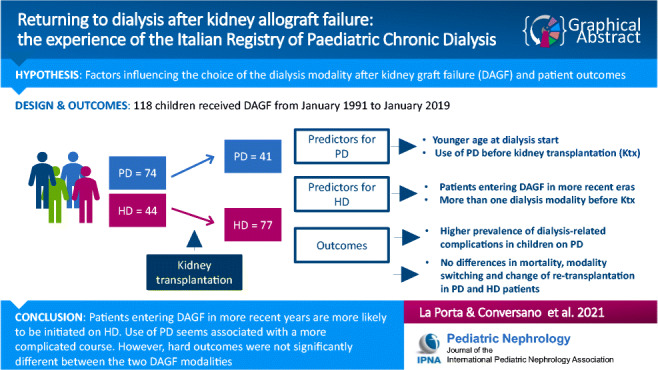

A higher resolution version of the Graphical abstract is available as Supplementary information

**Supplementary Information:**

The online version contains supplementary material available at 10.1007/s00467-021-05140-6.

## Introduction

Patients returning to dialysis after kidney allograft failure (DAGF) represent an emerging and challenging clinical problem for both the adult and the paediatric nephrology communities. Despite advances in kidney transplantation, long-term improvement in graft survival remained unchanged in Europe since 2000s [1], and DAGF is now included among the top five leading individual causes of dialysis initiation in adults [2]. In the 2019 Annual Data Report from the United States Renal Data System, patients returning to dialysis after kidney allograft failure (KAF) represent approximately 6% of the incident dialysis population and 15% of patients awaiting kidney transplantation [3]. Among DAGF patients, approximately 5% are under the age of 18 years, while up to 15% of transplanted paediatric patients have been reported as re-transplanted [4].

DAGF patients have historically been considered a high-risk population among dialysis patients. Increased mortality in adult patients on dialysis after KAF has been confirmed in some studies [5–8], but not in others [9–11], and conclusive evidence is lacking [12].

Furthermore, in this chronic kidney disease (CKD) patient population, the question of whether the dialysis modality has an impact on mortality has been also investigated [13], but, to date, there is no evidence of a clear advantage of one modality over another.

What is known for adults cannot be applied to children, mainly due to different underlying primary diseases, type of comorbidities, and dialysis complication rates*.* Paediatric CKD patients require prediction for a long-term kidney replacement therapy (KRT), thus representing a group with unique clinical problems.

Nevertheless, data about paediatric DAGF patients are very limited. A study conducted by the North American Pediatric Renal Trials and Collaborative Studies (NAPRTCS) registry investigated the mortality risk among DAGF and transplant-naïve dialysis children and showed no significant differences between the two patient groups [14]. No paediatric population studies have examined whether the dialysis modality initiated after transplantation affects mortality or whether and how the dialysis course performed before KAF influences DAGF outcomes.

Given the limited evidence on this topic in the paediatric setting, we aimed to analyse the clinical characteristics and outcomes of children with failed allografts returning to dialysis.

## Methods

Study data were obtained from the Italian Registry of Paediatric Chronic Dialysis (IRPCD), a nationwide dialysis network covering all the 12 Italian paediatric dialysis centres. For patient data to be collected by the IRPCD, patient consent must have been obtained.

We retrospectively evaluated data of patients < 18 years old returning to dialysis after graft failure, from January 1991 to January 2019, from the IRPCD. Patients who had access to the first transplant as pre-emptive (n = 7) were excluded because dialysis pre-transplant was a variable of interest for the primary outcome; therefore, data from 118 children were analysed.

The dialysis technique was defined as KRT at 30 days from its start. All new events regarding dialysis modality change, new onset of comorbidities, transplantation, or death are updated every 6 months in the Registry database. Every variation in dialysis modality accounted for a different cycle. Kidney transplant data were collected through a specific request for additional data from the participating dialysis centres.

We classified primary kidney diseases and causes of KAF according to the European Renal Association–European Dialysis and Transplantation Association (ERA–EDTA) definitions [15]. Complications were categorized as any health problem requiring hospitalization, unplanned visits, or surgical interventions. Dialysis-related complications were classified as those correlated explicitly to the dialysis treatment, such as malfunctioning of the dialysis access, peritonitis or dialysis access-related infection, hypotension due to excessive ultrafiltration, hypertension/fluid overload, and inadequate dialysis efficiency.

Information on each subject was updated to the last follow-up. Patients were followed until change in DAGF modality, death, or re-transplantation. Patients lost to follow-up before 18 years of age were censored. Patients who had transitioned to an adult nephrology centre were also censored at last follow-up.

The study aimed to describe the clinical characteristics of the cohort of children returning to dialysis after a KAF. We also intended to assess how a series of variables of interest conditioned the choice of post-transplantation dialysis modality. We eventually evaluated how the dialysis modality modified the hard outcomes recorded at the end of follow-up (death, re-transplantation, and switching dialysis modality).

### Statistical analysis

Frequencies and percentages described patient demographic and clinical characteristics at the time of transplantation for categorical variables and median and interquartile ranges for continuous variables. Chi-square or Fisher’s exact tests were used to evaluate potential differences in the categorical variables’ distribution according to the dialysis modality. Similarly, the Kruskal–Wallis test was used for continuous variables.

The potential predictive factors of the dialysis modality after transplantation that we investigated were gender, primary kidney disease, cause of KAF, dialysis modality before transplantation, number of dialysis cycles, comorbidity, duration of dialysis before transplantation, elapsed time between transplantation and dialysis, age and calendar year at transplantation. Information on all the variables considered was generally complete except for the cause of KAF (40% missing data). Hence, a complete-case analysis was performed excluding the cause of KAF from multivariable regression models. Univariate and multivariable analyses were conducted by logistic regression. Continuous variables were modeled as linear and then as restricted cubic splines with three knots fixed at their distribution tertiles. If there was no evidence of non-linear trends, the model with linear terms was chosen. The potential interactions between the variables included in the model were verified.

Follow-up data were available up to the end of June 2019. Outcomes of interest included patient death, re-transplantation, and change of dialysis modality. All analyses were performed in a competing risk setting [16] with time since transplantation as the primary timescale. Non-parametric cause-specific cumulative incidence function, i.e. the probability of that outcome occurring before either of the two competing events, was estimated for all three competing events. When we compared the dialysis modality, we performed a multivariable cause-specific hazards model, adjusted for potential confounders identified according to our a priori knowledge [17]. This modelling allows estimating the hazard corresponding to the cause-specific cumulative incidence function and, consequently, the hazard ratio (HR) of peritoneal dialysis (PD) vs. haemodialysis (HD) separately for each interest event. The following confounders were identified: gender, primary kidney disease, dialysis modality before transplantation, number of dialysis cycles, comorbidity, duration of dialysis before transplantation, age, and calendar year at transplantation. The proportionality assumption was checked by Schoenfeld residuals. Similarly to the prediction model, continuous variables were shaped first as linear and then as restricted cubic splines with three knots fixed at their distribution tertiles. If there was no evidence of non-linear trends, the model with linear terms was chosen. Potential modifications of the effect of dialysis modality were checked, including interaction terms with the model variables. Stata 13.0 was used for all statistical analyses.

## Results

### Patient characteristics

Baseline characteristics of the 118 patients who initiated dialysis after KAF during the study period are summarized in Table 1. The post-transplant dialysis choice was PD in 41 (35%) and HD in 77 (65%) patients, while before kidney transplantation, 74 (62.7%) had been on PD and 44 (37.2%) on HD. Of the 41 patients who underwent PD after KAF, 37 (90.2%) had been treated with PD before transplant, and 4 (9.8%) with HD. Among patients on HD after KAF, the pre-transplant dialysis modality distribution was HD in 40 (51.9%) and PD in 37 patients (48.1%). Primary kidney disease, cause of KAF, and age at dialysis re-initiation were significantly different between the two groups. After KAF, among patients treated with PD, there was a higher prevalence of HUS (9.8 vs. 2.6%) as primary kidney disease, while among patients treated with HD, a higher prevalence of cystic kidney diseases (16.9 vs. 2.4%) was observed. In the transplantation course, patients on PD after KAF were more prone to have had a primary disease recurrence (35 vs. 16%) and a chronic rejection (30 vs. 12%) than HD patients, among whom acute rejection was a more frequent cause of allograft loss. At dialysis re-initiation, compared with HD, patients on PD were younger (median age 11.5 years [IQR 7.3–16.2] vs. 14.6 years for HD [IQR 11.4–17.5]; p = 0.01), and re-started dialysis in earlier calendar years (median calendar year was 2004 [IQR 01–08] for PD patients vs. 2012 [IQR 07–17] for HD; p < 0.001). HD was more frequently adopted in patients with more than 2 dialysis cycles, and its choice depended primarily on patient and family preferences. Subjects who had undergone pre-transplant PD tended to continue in the same modality after KAF. Gender, donor type, the presence of comorbidities, and the length of dialysis pre- and post-transplant were comparable between the two groups.

**Table 1:** Baseline characteristics of the study cohort. (*CAKUT*, congenital anomalies of the kidneys and urinary tract; *HUS*, haemolytic uremic syndrome; *PTLD*, post-transplant lymphoproliferative disease)

	PD	HD	p
	N = 41 (35)	N = 77 (65)	
Gender (N (%)) (0 missing)			0.42
Male	23 (56.1)	49 (63.6)	
Female	18 (43.9)	28 (36.4)	
Primary kidney disease (N (%)) (0 missing)			0.05
CAKUT	15 (36.6)	32 (41.6)	
HUS	4 (9.8)	2 (2.6)	
Metabolic	2 (4.9)	3 (3.9)	
Cystic kidney disease	1 (2.4)	13 (16.9)	
Glomerulonephritis	15 (36.6)	25 (32.5)	
Ischemia	1 (2.4)	0 (0.0)	
Miscellaneous	3 (7.3)	2 (2.6)	
Comorbidity (N (%)) (0 missing)			0.12
No	31 (75.6)	67 (87.0)	
Yes	10 (24.4)	10 (13.0)	
Donor type (N (%)) (0 missing)			0.73
Deceased	40 (97.6)	75 (97.4)	
Living	1 (2.4)	2 (2.6)	
Cause of kidney allograft failure (N (%)) (48 missing)			0.01
Infection	2 (10.0)	1 (2.0)	
PTLD	0 (0.0)	1 (2.0)	
Thrombosis	1 (5.0)	3 (6.0)	
Acute rejection	0 (0.0)	10 (20.0)	
Chronic allograft nephropathy	3 (15.0)	20 (40.0)	
Chronic rejection	6 (30.0)	6 (12.0)	
Primary non-function	1 (5.0)	1 (2.0)	
Recurrence of primary disease	7 (35.0)	8 (16.0)	
Dialysis pre-transplant (N (%)) (0 missing)			<0.001
HD	4 (9.8)	40 (51.9)	
PD	37 (90.2)	37 (48.1)	
Dialysis cycle (N (%)) (4 missing)			0.05
2	36 (87.8)	47 (64.4)	
3	3 (7.3)	20 (27.4)	
4	2 (4.9)	5 (6.8)	
> 4	0 (0.0)	1 (1.4)	
Reason for dialysis modality choice (52 missing)			0.001
Difficulties in creating a vascular access	1 (5.3)	0 (0.0)	
Patient/family choice	1 (5.3)	20 (42.5)	
Patient age and/or size	0 (0.0)	1 (2.1)	
Peritoneal membrane failure	0 (0.0)	4 (8.5)	
Same modality previous cycle	17 (89.5)	19 (40.4)	
Social reasons	0 (0.0)	2 (4.2)	
Other	0 (0.0)	1 (2.1)	
Age at (median, IQR):			
Dialysis initiation pre-transplant (4 missing)	4.8 (2.0;6.5)	5.9 (2.3;11.3)	0.05
Kidney transplant (0 missing)	6.5 (4.2;9.7)	7.9 (5.1;13.9)	0.05
Dialysis re-initiation post-transplant (0 missing)	11.5 (7.3;16.2)	14.6 (11.4;17.5)	0.01
Calendar year at (median, IQR):			
Dialysis initiation pre-transplant (4 missing)	1998 (91;04)	2004 (97;09)	< 0.001
Kidney transplant (0 missing)	2000 (92;05)	2007 (00;11)	< 0.001
Dialysis re-initiation post-transplant (0 missing)	2004 (01;08)	2012 (07;17)	< 0.001
Pre-transplant dialysis duration (months) (median, IQR) (4 missing)	17.8 (8.7;31.6)	22.5 (12.8;33.7)	0.30
Time between transplant and dialysis re-initiation (months) (median, IQR) (0 missing)	33.6 (6.7;82.2)	56.4 (17.1;100.3)	0.26
Time to graft failure (0 missing)			0.21
Early (< 1 year)	14 (34.1)	18 (23.4)	
Late (> = 1 year)	27 (65.9)	59 (76.6)	

Univariate and multivariable analyses were conducted to evaluate factors that influenced the type of post-transplant dialysis (Table 2). In the multivariable analysis, the probability of being treated with PD after KAF was higher for patients who underwent PD in the pre-transplant dialysis cycle (OR 8.20; 95%CI 1.82–37.01; p = 0.006) and in the less recent era (OR 0.87 per year increase; 95% CI 0.80–0.94; p = 0.001). Gender, age, primary kidney disease, presence of comorbidity, dialysis vintage, and transplant duration did not affect the choice of dialysis modality.

**Table 2:** Odds ratios and 95% confidence intervals of the risk of being treated with PD compared to HD estimated by univariate and multivariable logistic regression (N = 111) (*CAKUT*, congenital anomalies of the kidneys and urinary tract; *HUS*, haemolytic uremic syndrome)

	Univariate analysis		Multivariable analysis	
	OR	95% CI	OR	95% CI
Gender				
Male	1.00	ref	1.00	ref
Female	1.33	0.61; 2.92	1.21	0.38; 3.84
Primary kidney disease				
CAKUT	1.00	ref	1.00	ref
Glomerulonephritis	1.20	0.48; 2.97	2.14	0.63; 7.29
HUS/ischemica	10.00	1.06; 94.01	9.16	0.44; 188.34
Miscellaneous	0.71	0.23; 2.19	0.98	0.22; 4.38
Dialysis cycle				
2	1.00	ref	1.00	ref
3+	0.28	0.10; 0.81	0.56	0.12; 2.59
Dialysis pre-transplant				
HD	1.00	ref	1.00	ref
PD	10.36	3.34; 32.19	8.20	1.82; 37.01
Comorbidity				
No	1.00	ref	1.00	ref
Yes	2.30	0.84; 6.24	1.51	0.38; 6.06
Age at dialysis post-transplant				
Unit increase	0.88	0.81; 0.96	0.85	0.72; 1.00
Calendar year at dialysis post-transplant				
Unit increase	0.89	0.84; 0.94	0.87	0.80; 0.94
Pre-transplant dialysis duration (months)				
Unit increase	1.00	0.98; 1.01	0.99	0.96; 1.01
Time between transplant and dialysis re-initiation (months)				
Unit increase	1.00	0.99; 1.00	1.00	0.99; 1.02

### Outcome data

**Fig. 1:**
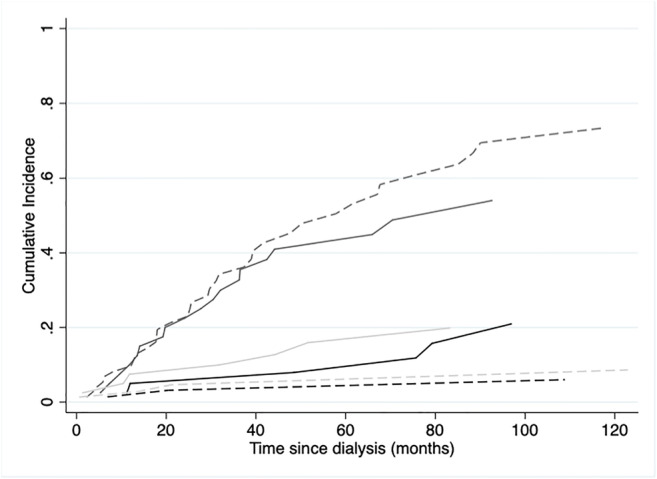
Non-parametric cumulative incidence for the competing events stratified by dialysis modality after kidney allograft failure. PD patients, solid line; HD patients, dashed lines. Death, black; Transplantation, grey; Switching modality, light grey. Gray’s test for equality of the cumulative curves: p = 0.09 for death, p = 0.19 for transplantation, p = 0.24 for switching modality

At the end of follow-up, 6 PD (13.6%) and 3 HD (4.2%) patients died, with a cumulative incidence of mortality slightly higher in PD patients (p = 0.09) (Fig. 1). However, after adjustment for several covariates, patients on PD exhibited an increased but non-significant risk of mortality compared with HD (HR 2.15; 95% CI 0.54–8.6; p = 0.28) (Table 3).

**Table 3:** Hazard ratio with 95% confidence intervals for the risk of death estimated by multivariable Fine and Gray model (N = 104) (*CAKUT*, congenital anomalies of the kidneys and urinary tract; *HUS*, haemolytic uremic syndrome)

Dialysis post-transplant	HR	95% CI
HD	1.00	ref
PD	3.52	0.23; 53.92
Gender		
Male	1.00	ref
Female	0.76	0.11; 5.13
Primary kidney disease		
CAKUT	1.00	ref
Glomerulonephritis	1.00	0.19; 95.21
HUS/ischemic	7.01	0.42; 116.92
Miscellaneous	0.52	0.03; 9.33
Dialysis pre-transplant		
HD	1.00	ref
PD	0.32	0.02; 4.72
Dialysis cycle		
2	1.00	ref
3+	0.99	0.06; 15.99
Comorbidity		
No	1.00	ref
Yes	1.53	0.17; 13.83
Pre-transplant dialysis duration (months)		
Unit increase	0.98	0.94; 1.03
Age at dialysis post-transplant		
Unit increase	0.95	0.78; 1.17
Calendar year at dialysis post-transplant		
Unit increase	0.96	0.94; 1.03

Seven out of 41 PD patients (17%) and 4 out of 77 HD individuals (5%) required a switching in dialysis modality; 19 patients on PD (46%) and 36 on HD (47%) received a re-transplantation. Cumulative incidence for both events at the end of the follow-up was similar. Multivariable analysis risk of re-transplantation and switching modality are shown in Supplementary Table 1.

### Analysis of complications

Overall, 86 (73%) and 55 (46.7%) patients had no complications in the pre- and post-transplant dialysis course, respectively. Sixty complications in 74 PD patients (resulting in 1 episode per 33.4 patient-months) and 19 complications in 44 HD patients (resulting in 1 episode per 49.8 patient-months) were reported in the pre-transplant course. One hundred four complications in 41 PD patients (resulting in 1 episode per 17.4 patient-months) and 90 complications in 77 HD patients (resulting in 1 episode per 31.4 patient-months) were reported in the DAGF course. Rate of complications occurring during the pre- and post-transplant period, based on dialysis modality, are reported in Table 4. DAGF patients on PD had a higher rate of both clinical and dialysis-related complications as compared with those on HD. Among patients who re-started PD after KAF, dialysis-related complications were peritonitis and exit-site infections (9 patients; 45%), peritoneal membrane failure (5; 25%), encapsulating peritoneal sclerosis (3; 15%), hypertension (2; 10%), and mechanical complications (1; 5%). In the HD cohort, dialysis-related complications in the post-transplant course were arteriovenous fistula or catheter malfunction (5; 35.8%), severe fluid overload (3; 21.4%), hypertension (2; 14.3%), catheter exit-site infection (2; 14.3%), intradialytic hypotension (1; 7.1%), and unknown causes (1; 7.1%). A comparison in the type of dialysis-related complications in pre- and post-transplantation period is reported in Supplementary Table 2. In those patients who had dialysis-related complications in the pre-transplant course, the relative risk for complications in the dialysis course after KAF was 1.16 (95% CI 0.53–2.53) for PD and 6.15 (95% 2.57–14.7) for HD.

**Table 4:** Complications in pre- and post-transplantation period according to dialysis modality

	PD (pre-transplant)	HD (pre-transplant)	p
	N = 74	N = 44	
Pre-transplant complications (overall)			0.106
0	49 (66.2)	37 (84.1)	
1	8 (10.8)	2 (4.5)	
2+	17 (23)	5 (11.4)	
Pre-transplant complications related to dialysis			0.147
0	57 (77)	40 (91)	
1	6 (8.1)	2 (4.5)	
2+	11 (14.9)	2 (4.5)	
	PD (post-transplant)	HD (post-transplant)	
	N = 41	N = 77	
Post-transplant complications (overall)			0.002
0	12 (29.3)	43 (55.8)	
1	6 (14.6)	16 (20.8)	
2+	23 (56.1)	18 (23.4)	
Post-transplant complications related to dialysis			0.002
0	21 (51.2)	63 (81.8)	
1	9 (22)	6 (7.8)	
2+	11 (26.8)	8 (10.4)	

## Discussion

This study reports the first comparison of dialysis modalities in a nationally representative DAGF paediatric population. Based on data collected by the IRPCD, we investigated factors influencing the choice of the dialysis modality after KAF and evaluated hard outcomes of DAGF patients.

In our series, the probability of being prescribed with PD after KAF was significantly higher in patients treated with the same modality before transplantation and in the earliest era. While PD still represents the incident modality of choice for children with CKD stage 5, our findings seem to indicate that HD has become in recent years the preferred modality in DAGF patients. HD practices for children have improved over the past 20 years, especially because of technological developments and the evolution from an “adequate” to an “optimum” dialysis prescription [18]. The morbidity of the sessions has decreased, and this has simplified the diffusion of extracorporeal therapies in most of the paediatric nephrology units. Children receiving DAGF might be considered an at-risk population as compared to transplant- and dialysis-naïve patients, because of previous courses of immunosuppressants, increased comorbidities, and higher metabolic needs. Overall, this might justify the prevalent use of HD over PD, especially in more recent years and in those patients who have received several previous dialysis courses.

In a previous study, the NAPRTCS network analysed the survival rate in DAGF and in transplant naïve children, observing no differences between these two groups [14]. Conversely, among adult DAGF patients, a tendency towards an increased morbidity and mortality rate has been reported [5–8]. Causes of increased mortality in DAGF adult patients have been previously investigated in several studies, separately considering immunological and non-immunological factors, as well as factors related to transplantation, dialysis modality, and dialysis access [19, 20].

In our paediatric DAGF population, we report a trend towards an increased but non-significant mortality risk among patients on long-term PD compared with those on HD. This issue is consistent with a previous study we conducted on the IRPCD data. In a cohort of propensity-matched incident dialysis patients, long-term PD treatment was associated with an increased risk of death [21]. Chesnaye et al. compared the mortality risk in a propensity-matched population of European children with CKD stage 5 and showed that patients selected to start HD had an increased mortality risk compared with those on PD, but especially during the first year of dialysis and when starting at older than 5 years [22]. In a series of 16,113 adult patients on DAGF, Perl et al. demonstrated that, compared with HD, PD is associated with an early survival advantage, inferior late survival, and similar overall survival [23]. Overall, the comparison between dialysis modalities in the adult population resulted in conflicting results, and there is not enough evidence to support the use of a specific modality in DAGF patients [12].

Several reasons might explain the different mortality risk over time according to DAGF modality, including a selection bias for the initial modality. Indeed, patients who require emergency dialysis are more frequently treated with HD, and this may partially explain the more favorable survival outcomes of PD during the first DAGF period. Conversely, several studies correlated the worse long-term survival outcome of PD with the loss of function of the peritoneal membrane, mainly related to frequent peritonitis and dialysis duration. Dialysis vintage, kidney transplant, and calcineurin inhibitor use have been indicated as risk factors for the development of encapsulating peritoneal sclerosis, which is associated with a high mortality rate [24].

In a NAPRTCS registry study, Chen et al. showed a slightly increased infection risk for PD in a DAGF patient cohort compared to transplant-naïve patients [25]. Frequent peritonitis can lead to peritoneal membrane failure and inadequate dialysis associated with worsening of the patient’s clinical conditions, increased cardiovascular morbidity, and the frequent need to change the dialysis modality [26]. In our study, infections and inadequate dialysis efficiency due to peritoneal membrane failure or encapsulating peritoneal sclerosis were the most important complications in DAGF children on PD. However, despite the significant higher percentage of both clinical and dialysis-related complications in children on PD compared to HD after KAF, the more complicated course was not associated with a higher cumulative incidence of switching dialysis in patients on PD.

Our study has some limitations, mainly concerning its retrospective nature. Information on some of the analysed variables was not available for all patients, especially regarding complications. Moreover, using the registry database, we have been unable to collect important information about type and number of dialysis accesses, and more detailed data on the transplant course (type of immunosuppressants and the timing of their withdrawal). On the other hand, our study reports on data collected nationally through an established network that includes all the 12 paediatric dialysis centres active in the country.

In conclusion, this study is one of the first to analyse the emerging population of paediatric DAGF patients and to compare the results by dialysis modality. Our results show that after KAF, patients tended to start dialysis on the same modality adopted before kidney transplant and that patient/family preference was the main reason for changing modality. Older patients and those entering DAGF in more recent years were more likely to be initiated on HD rather than PD. The use of PD seems associated with a more complicated course in children initiating DAGF. However, hard outcomes, including mortality, switching dialysis modality and the probability of receiving a second transplant, were not significantly different between the two DAGF modalities, while there was a trend to an increased mortality risk among patients treated with PD in the long-term. Further research is needed to evaluate the effect of immunosuppressive therapy, kidney graft nephrectomy, panel reactive antibody levels, and timing of re-transplantation on the outcomes of paediatric patients undergoing DAGF.

## Supplementary Information

ESM 1 (PDF 54 kb)

ESM 2 Graphical abstract (PPTX 46.8 kb)

## References

[1] Coemans M, Süsal C, Döhler B, Anglicheau D, Giral M, Bestard O, Legendre C, Emonds MP, Kuypers D, Molenberghs G, Verbeke G, Naesens M (2018). Analyses of the short- and long-term graft survival after kidney transplantation in Europe between 1986 and 2015. Kidney Int.

[2] Perl J, Hasan O, Bargman JM, Jiang D, Na Y, Gill JS, Jassal SV (2011). Impact of dialysis modality on survival after kidney transplant failure. Clin J Am Soc Nephrol.

[3] United States Renal Data System (2019). 2019 USRDS Annual Data Report: Epidemiology of kidney disease in the United States.

[4] Rao PS, Schaubel DE, Jia X, Li S, Port FK, Saran R (2007). Survival on dialysis post-kidney transplant failure: results from the Scientific Registry of Transplant Recipients. Am J Kidney Dis.

[5] Heaphy EL, Poggio ED, Flechner SM, Goldfarb DA, Askar M, Fatica R, Srinivas TR, Schold JD (2014). Risk factors for retransplant kidney recipients: relisting and outcomes from patients’ primary transplant. Am J Transplant.

[6] Beltrán S, Gavela E, Kanter J, Sancho A, Avila A, Górriz JL, Crespo JF, Pallardó LM (2009). Beginning hemodialysis: do patients with a failed renal transplant start in worse condition?. Transplant Proc.

[7] Bisigniano L, Laham G, Giordani MC, Tagliafichi V, Hansen Krogh D, Maceira A, Rosa-Diez GJ (2020). Reduced survival in patients who return to dialysis after kidney allograft failure. Clin Transplant.

[8] Brar A, Markell M, Stefanov DG, Timpo E, Jindal RM, Nee R, Sumrani N, John D, Tedla F, Salifu MO (2017). Mortality after renal allograft failure and return to dialysis. Am J Nephrol.

[9] Rao PS, Schaubel DE, Saran R (2005). Impact of graft failure on patient survival on dialysis: a comparison of transplant-naïve and post-graft failure mortality rates. Nephrol Dial Transplant.

[10] Varas J, Pérez-Sáez MJ, Ramos R, Merello JI, de Francisco ALM, Luño J, Praga M, Aljama P, Pascual J, Optimizing Results in Dialysis (ORD) group (2019). Returning to haemodialysis after kidney allograft failure: a survival study with propensity score matching. Nephrol Dial Transplant.

[11] Mourad G, Minguet J, Pernin V, Garrigue V, Peraldi MN, Kessler M, Jacquelinet C, Couchoud C, Duny Y, Daurès JP (2014). Similar patient survival following kidney allograft failure compared with non-transplanted patients. Kidney Int.

[12] Kabani R, Quinn RR, Palmer S, Lewin AM, Yilmaz S, Tibbles LA, Lorenzetti DL, Strippoli GF, McLaughlin K, Ravani P, Network AKD (2014). Risk of death following kidney allograft failure: a systematic review and meta-analysis of cohort studies. Nephrol Dial Transplant.

[13] Kang GW, Jang MH, Hwang EA, Park SB, Han SY (2013). Comparison of peritoneal dialysis and hemodialysis after kidney transplant failure. Transplant Proc.

[14] Chen A, Martz K, Kershaw D, Magee J, Rao PS (2010). Mortality risk in children after renal allograft failure: a NAPRTCS study. Pediatr Nephrol.

[15] Kramer A, Boenink R, Noordzij M, Bosdriesz JR, Stel VS, Beltrán P, Ruiz JC, Seyahi N, Comas Farnés J, Stendahl M, Garneata L, Winzeler R, Golan E, Lopot F, Korejwo G, Bonthuis M, Lassalle M, Slon Roblero MF, Kuzema V, Hommel K, Stojceva-Taneva O, Asberg A, Kramar R, Hemmelder MH, De Meester J, Vazelov E, Andrusev A, Castro de la Nuez P, Helve J, Komissarov K, Casula A, Magaz Á, Santiuste de Pablos C, Bubić I, Traynor JP, Ioannou K, Idrizi A, Palsson R, des Grottes JM, Spustova V, Tolaj-Avdiu M, Jarraya F, Nordio M, Ziginskiene E, Massy ZA, Jager KJ (2020). The ERA-EDTA Registry Annual Report 2017: a summary. Clin Kidney J.

[16] Fine JP, Gray RJ (1999). A proportional hazards model for the subdistribution of a competing risk. J Am Stat Assoc.

[17] Noordzij M, Leffondré K, van Stralen KJ, Zoccali C, Dekker FW, Jager KJ (2013). When do we need competing risks methods for survival analysis in nephrology?. Nephrol Dial Transplant.

[18] Fischbach M, Edefonti A, Schröder C, Watson A, European Pediatric Dialysis Working Group (2005). Hemodialysis in children: general practical guidelines. Pediatr Nephrol.

[19] Gill JS, Abichandani R, Kausz AT, Pereira BJG (2002). Mortality after kidney transplant failure: the impact of non-immunologic factors. Kidney Int.

[20] Park JT, Yoo TH, Chang TI, Lee JH, Lee DH, Kim BS, Kang SW, Lee HY, Kim MS, Kim SI, Kim YS, Choi KH (2010). Predictors of mortality in patients returning to dialysis after allograft loss. Blood Purif.

[21] Vidal E, Chesnaye NC, Paglialonga F, Minale B, Leozappa G, Giordano M, Gianoglio B, Corrado C, Roperto RM, Chimenz R, Mencarelli F, Ratsch IM, Murer L, Verrina E, Italian Registry for Paediatric Chronic Dialysis (2018). A propensity-matched comparison of hard outcomes in children on chronic dialysis. Eur J Pediatr.

[22] Chesnaye NC, Schaefer F, Groothoff JW, Bonthuis M, Reusz G, Heaf JG, Lewis M, Maurer E, Paripović D, Zagozdzon I, van Stralen KJ, Jager KJ (2016). Mortality risk in European children with end-stage renal disease on dialysis. Kidney Int.

[23] Perl J, Dong J, Rose C, Jassal SV, Gill JS (2013). Is dialysis modality a factor in the survival of patients initiating dialysis after kidney transplant failure?. Perit Dial Int.

[24] Vidal E, Edefonti A, Puteo F, Chimenz R, Gianoglio B, Lavoratti G, Leozappa G, Maringhini S, Mencarelli F, Pecoraro C, Ratsch IM, Cannavò R, De Palo T, Testa S, Murer L, Verrina E, Italian Registry of Pediatric Chronic Dialysis (2013). Encapsulating peritoneal sclerosis in paediatric peritoneal dialysis patients: the experience of the Italian Registry of Pediatric Chronic Dialysis. Nephrol Dial Transplant.

[25] Chen A, Martz K, Rao PS (2012). Does allograft failure impact infection risk on peritoneal dialysis: a North American Pediatric Renal Trials and Collaborative Studies Study. Clin J Am Soc Nephrol.

[26] Verrina E, Edefonti A, Gianoglio B, Rinaldi S, Sorino P, Zacchello G, Lavoratti G, Maringhini S, Pecoraro C, Calevo MG, Turrini Dertenois L, Perfumo F (2004). A multicenter experience on patient and technique survival in children on chronic dialysis. Pediatr Nephrol.

