# Supplementary material for: Returning to dialysis after kidney allograft failure: the experience of the Italian Registry of Paediatric Chronic Dialysis
Source: Pediatr Nephrol. 2021 Jun 14;36(12):3961–9. doi: 10.1007/s00467-021-05140-6 (PMC8599402; doi:10.1007/s00467-021-05140-6)
Supplement: Supplementary file 1 — (PDF 54 kb) [file 467_2021_5140_MOESM1_ESM.pdf]

Online supplementary material

Returning to Dialysis After Kidney Allograft Failure: The Experience of the Italian Registry of Paediatric Chronic Dialysis

Journal: Pediatric Nephrology

Edoardo La Porta, Ester Conversano, Daniela Zugna, Roberta Camilla, Raffaella Labbadia, Fabio Paglialonga, Mattia Parolin, Enrico Vidal and Enrico Verrina, on behalf of the Italian Registry of Paediatric Chronic Dialysis

Corresponding author:
Enrico Vidal
Paediatric Nephrology Program
Division of Paediatrics – Department of Medicine (DAME)
University of Udine
P.le S.M della Misericordia, 15
33100 Udine – ITALY
Email: enrico.vidal@inwind.it

Supplementary Table 1. Hazard ratio with 95% confidence intervals for the risk of switching modality and transplantation estimated by multivariable Fine and Gray model (N=104).

	**Switching dialysis modality**		**Transplantation**	
	**HR**	**95% confidence interval**	**HR**	**95% confidence interval**
**Dialysis post-transplant**				
HD	1.00	ref	1.00	ref
PD	2.20	0.38;12.54	0.59	0.26;1.34
**Gender**				
Male	1.00	ref	1.00	ref
Female	1.87	0.42;8.25	1.43	0.73;2.80
**Primary kidney disease**				
CAKUT	1.00	ref	1.00	ref
Glomerulonephritis	2.27	0.49;10.39	0.97	0.50;1.89
HUS / Ischemic	-	-	1.76	0.35;8.85
Miscellaneous	2.35	0.31;17.84	1.55	0.66;3.65
**Dialysis pre-transplant**				
HD	1.00	ref	1.00	ref
PD	2.19	0.29;16.51	1.03	0.48;2.24
**Dialysis cycle**				
2	1.00	ref	1.00	ref
3+	0.96	0.14;6.56	1.25	0.55;2.84
**Comorbidity**				
No	1.00	ref	1.00	ref
Yes	0.47	0.06;3.54	1.32	0.57;3.07
**Pre-transplant dialysis duration (months)**				
Unit increase	1.01	0.99;1.03	0.98	0.96;1.00
**Age at dialysis post-transplant**				
Unit increase	0.95	0.82;1.11	0.98	0.91;1.05
**Calendar year at dialysis post-transplant**				
Unit increase	1.01	0.92 ;1.11	1.01	0.96 ;1.05

Supplementary table 2. Frequencies of patients with dialysis-related complications in pre- and post-transplant period according to dialysis modality.

	PD		HD	
**Type of complication**	**Pre-transplant course (n=74)**	**Post-transplant course (n=41)**	**Pre-transplant course (n=44)**	**Post-transplant course (n=77)**
Peritonitis or dialysis access-related infection	8 (47%)	9 (45%)	3 (75%)	2 (14.4%)
Malfunctioning of dialysis access (mechanical)	1 (5.9%)	1 (5%)	-	5 (35.7%)
Inadequate dialysis efficiency	2 (11.8%)	8 (40%)	-	-
Hypertension/fluid overload	3 (17.6%)	2 (10%)	1 (25%)	5 (35.7%)
Hypotension	2 (11.8%)	-	-	1 (7.1%)
Other	1 (5.9%)	-	-	-
Unknown	-	-	-	1 (7.1%)
