# Supplementary material for: Returning to dialysis after kidney allograft failure: the experience of the Italian Registry of Paediatric Chronic Dialysis
Source: Pediatr Nephrol. 2021 Jun 14;36(12):3961–9. doi: 10.1007/s00467-021-05140-6 (PMC8599402; doi:10.1007/s00467-021-05140-6)
Supplement: Supplementary file 2 — Graphical abstract (PPTX 46.8 kb) [file 467_2021_5140_MOESM2_ESM.pptx]

## Slide 1
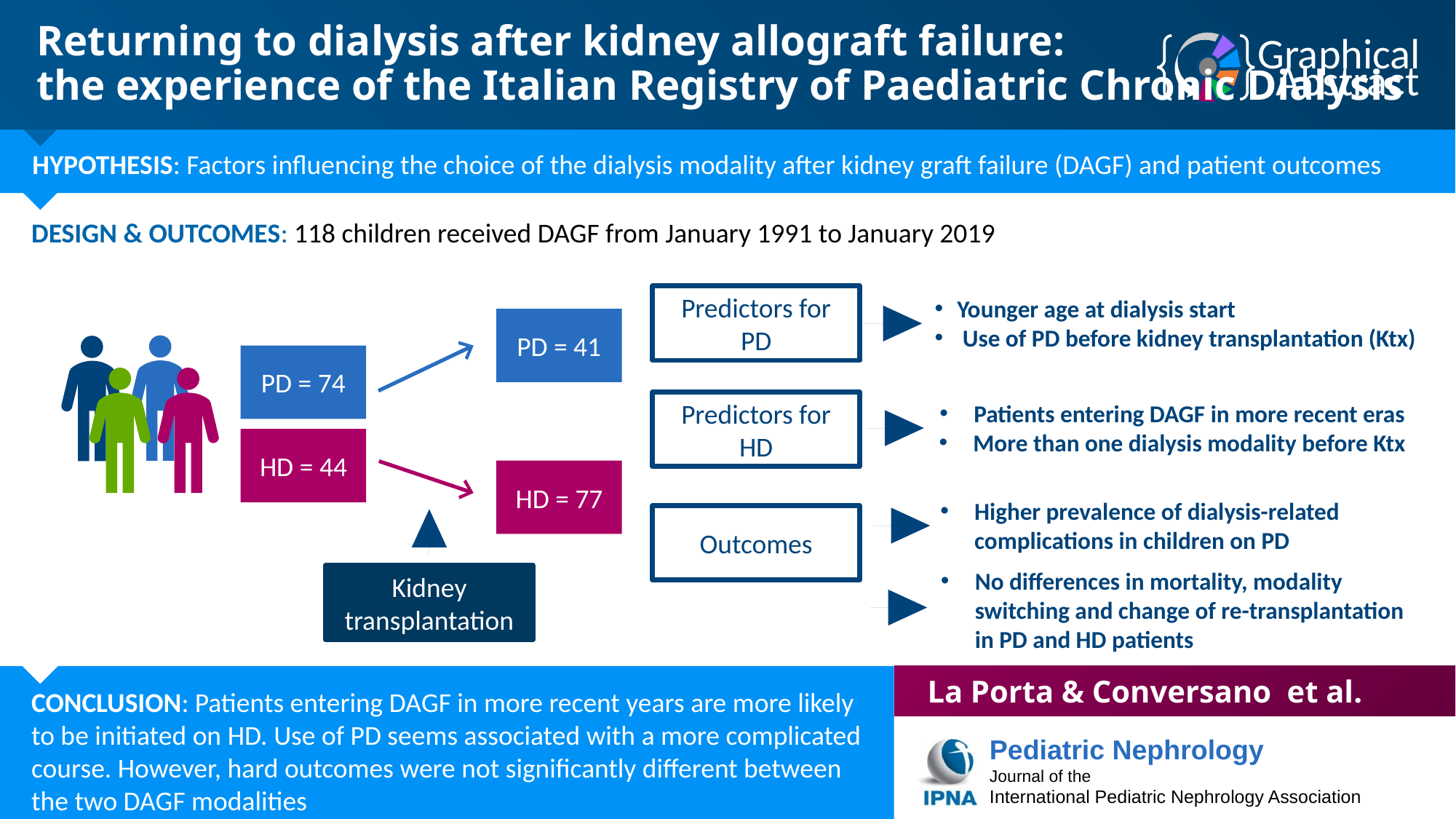

Returning to dialysis after kidney allograft failure:
the experience of the Italian Registry of Paediatric Chronic Dialysis
HYPOTHESIS: Factors influencing the choice of the dialysis modality after kidney graft failure (DAGF) and patient outcomes
DESIGN & OUTCOMES: 118 children received DAGF from January 1991 to January 2019
Predictors for PD
Younger age at dialysis start
 Use of PD before kidney transplantation (Ktx)
PD = 41
PD = 74
Predictors for HD
Patients entering DAGF in more recent eras
More than one dialysis modality before Ktx
HD = 44
HD = 77
Higher prevalence of dialysis-related complications in children on PD
Outcomes
No differences in mortality, modality switching and change of re-transplantation in PD and HD patients
Kidney transplantation
La Porta & Conversano et al. 2021
CONCLUSION: Patients entering DAGF in more recent years are more likely to be initiated on HD. Use of PD seems associated with a more complicated course. However, hard outcomes were not significantly different between the two DAGF modalities
